# Comparison of clinical and biological characteristics of HIV-infected patients presenting *Cryptococcus neoformans* versus *C. curvatus/C. laurentii* meningitis

**DOI:** 10.1186/s12879-021-06849-3

**Published:** 2021-11-15

**Authors:** Bive Zono, Michel Moutschen, Hippolyte Situakibanza, Rosalie Sacheli, Gaultier Muendele, Pius Kabututu, Adolphe Biakabuswa, Nicole Landu, Georges Mvumbi, Marie-Pierre Hayette

**Affiliations:** 1 Molecular Biology Service, Department of Basic Sciences, Faculty of Medicine, University of Kinshasa, Kinshasa, Democratic Republic of Congo; 2 Department of Infectious Diseases and General Internal Medicine, University Hospital Center of Liege, Liege, Belgium; 3 Infectious Diseases Service, Department of Internal Medicine/Department of Tropical Medicine, Faculty of Medicine, University of Kinshasa, Kinshasa, Democratic Republic of Congo; 4 National Reference Center for Mycosis, University Hospital Center of Liege, Liege, Belgium; 5 Advanced HIV Infection Management Unit, Internal Medicine Department, Centre Hospitalier Mère et Enfant de NGABA, Kinshasa, Democratic Republic of Congo; 6 Advanced HIV Infection Management Unit, Internal Medicine Department, Centre Médical et Evangélique Révérend LUYINDU, Kinshasa, Democratic Republic of Congo; 7 Center for Interdisciplinary Research on Medicines, University of Liege, Liege, Belgium

**Keywords:** *Cryptococcus neoformans*, *Cryptococcus curvatus*, *Cryptococcus laurentii*, Meningitis, HIV, Clinical characteristic, Biological characterization, Kinshasa, DRC

## Abstract

**Background:**

Cryptococcal meningitis is mainly caused by *Cryptococcus neoformans/C. gattii* complex. We compared the clinical, biological, and antifungal susceptibility profiles of isolates from HIV-Infected Patients (HIVIP) with *C. neoformans* (*Cn*) versus *C. curvatus/C. laurentii* (*Cc/Cl*) meningitis.

**Methods:**

Comparative analytical study were conducted. Apart from patients’ clinical data, the following analysis were performed and the results were compared in both groups: biochemical examination, cryptococcal antigen test, India ink staining, and culture on Cerebral Spinal Fluid (CSF), strains identification by mass spectrometry, ITS sequencing, PCR serotyping and antifungal susceptibility. The main outcome variable was the “species of *Cryptococcus* identified”, which was compared to other variables of the same type using the Pearson Chi-square test or the Fisher exact test.

**Results:**

A total of 23 (79.3%) *Cn* meningitis cases versus 6 (20.7%) *Cc/Cl* meningitis were retained*.* *Cn* meningitis was more frequently associated with headache (100% vs 50%, *p* = 0.005) than *Cc/Cl* meningitis and meningeal signs were more frequent in *Cn* infected patients. Biologically, hypoglycorrhachia and low CD_4_ count were more observed in *Cn* group (90% vs 20% of patients, *p* = 0.01; 45.6 vs 129.8 cells/µL, *p* = 0.02, respectively). A higher proportion of *Cn* strains (91.3%) showed a low Minimum Inhibitory Concentration (MIC) (< 8 mg/L) for fluconazole compared to *Cc/Cl* strains (66.7%). Also, *Cc/Cl* strains resistant to 5-flucytosine and amphotericin B were found in 16.7% of cases for each of the two antifungal agents. *Cryptococcus* detection by routine analysis (India ink, culture, and antigens) was better for *Cn* samples than *Cc/Cl*. Except ITS sequencing, which identified all strains of both groups, mass spectrometry and serotyping PCR identified *Cn* strains better than *Cc/Cl* (100% vs 80%, *p* = 0.1; 100% vs 0%, *p* < 0.0001, respectively). After treatment with amphotericin B, 5-flucytosine, and fluconazole in both groups, the outcome was similar.

**Conclusions:**

Clinical presentation of *Cn* meningitis is certainly more severe than that of *Cc/Cl* meningitis, but *Cc/Cl* infection should be considered in the management of HIVIP with meningeal syndrome because of the diagnostic difficulty and the high MICs of antifungal agents required for the treatment of meningitis due to these cryptococcal species.

## Background

In clinical pathology, two species of *Cryptococcus* spp. are mainly involved in Meningeal Cryptococcosis (MC), namely *Cryptococcus neoformans *(*Cn*) and *C. gattii *(*Cg*) [1]*.* The interest of *Cryptococcus* spp. identification down to species level is based on the fact that some species such as *C. gattii* cause infections that require a much more intensive therapeutic approach than those caused by *C. neoformans* [2]. Non*-neoformans* and non*-gattii* species have long been considered as saprophytes and non-pathogenic to humans. However, the prevalence of opportunistic infections due to these species (involving the skin, lungs, bloodstream, and central nervous system) has been increasing all over the world in recent years. Among these species, *C. laurentii* and *C. albidus* are implicated in 80% of cases [3, 4]. Also in rare cases, *C. curvatus* has been incriminated in peritoneal and myeloradicular infections in hospital settings [5–7]. Few studies are comparing different characteristics of infections caused by the *C. neoformans/C. gattii* complex versus those due to non-*neoformans* and non-*gattii* species in HIV-Infected Patients (HIVIP).

We hypothesized that the clinical and biological characteristics of HIVIP with meningitis due to *Cn* could be different from those of patients infected by *C. curvatus/C. laurentii* (*Cc/Cl*)*.* The objective of this study was to compare the clinical, biological, and therapeutic characteristics, antifungal susceptibility profile of strains isolated from HIVIP with *Cn* versus *Cc/Cl* meningitis. In addition, the molecular identification of the strains was also compared.

## Methods

### Study design, patients, and samples

This is a comparative analytical study. The patients were drawn from a cross-sectional study conducted in Kinshasa (Democratic Republic of Congo) from 1st February 2019 to 29th February 2020, having included HIVIP with the meningeal syndrome in the hospitals with expertise in the management of advanced HIV-infection, namely: Centre Hospitalier Mère et Enfant de NGABA (CHME NGABA), Centre Médical et Evangélique Révérend LUYINDU (CME LUYINDU) and Centre Hospitalier Roi Baudouin 1^er^ (CHRB 1^er^). The overall data from this study have not yet been published. In these public hospitals supported by Doctors without Borders-Belgium (MSF), lumbar puncture in HIVIP has specific indications. It was performed in each patient presenting meningeal signs or neurological symptoms including headache, epilepsy, lethargy, or cognitive deficits and/or in all HIVIP with a CD_4_ LT number < 100 cells/µL with cryptococcal antigenemia. Diagnostic confirmation of cryptococcosis is based on the blood cryptococcal antigen detection for serum cryptococcosis or the Cerebral Spinal Fluid (CSF) cryptococcal antigen detection for MC. In the present study, routine diagnostic confirmation was extended. Thus, MC diagnosis was based on the detection in the patient CSF, of cryptococcal antigens and/or the yeasts by India ink staining and/or positive culture.

### Initial analysis

The detection of Cryptococcal Antigens (CrAg) was carried out in the CSF of each patient, using the CrAg LFA IMMY test (Immuno-mycologic, Norman, OK, USA). Direct staining with India ink on the CSF was also carried out to reveal the cryptococcal capsule and the CSF was cultured on Sabouraud Dextrose Agar-Chloramphenicol medium (SDA-C, bioMérieux, France) at 30 °C for 48 to 72 h. Proteinorachia was determined by the Pandy test, a previously described qualitative test [8].

### Identification by MALDI-TOF MS

The MALDI-TOF MS (Matrix-Assisted Laser Desorption Ionization Time-Of-Flight Mass Spectrometry, Bruker Daltonics GmbH, Germany), was used for the identification of all fungal strains at CHU de Liège (Belgium). From the culture on SDA-C, an extended direct deposit was performed by adding 1 μL of 70% formic acid to the sample on a MALDI target plate (MSP 96 BC ground steel target; Bruker Daltonics). Then, 1 μL of saturated cyano-4-hydroxycinnamic acid solution (HCCA matrix; Bruker Daltonics) was added. A Bruker Bacterial Test Standard (BTS255343; Bruker Daltonics) was used for instrument calibration. Each microorganism tested was spotted twice on the same target slide. Measurement was performed with MALDI Flex control V3.4 (Bruker Daltonics) following the settings suggested by the manufacturer using automated collecting spectra. The spectra of each duplicated spot were compared with those in the reference library (BD 8326 or version V 9.0) [9]. The following score was considered for the identification of the fungal species: MS Score ≥ 1.5 and the three first results identical and consistent with the appearance of the colonies on agar.

### Molecular analysis

#### DNA extraction

Genomic DNA was extracted from the fresh 24-h cultures on SDA-C using the NucleoSpin blood quick pure kit (Macherey-Nagel, Duren, Germany). Two preliminary steps were added to the manufacturer’s protocol, namely bead-beating, and thermal shock. In a 2 mL tube containing 0.5 mm glass beads (Roche Diagnostics GmbH, Penzberg, Germany), colonies were mixed with 350 µL lysis buffer (Promega Corporation, USA). The mixture was vortexed five times at 6000 vibrations per minute for 40 s (bead-beating). Between each pass, the tube was cooled between − 20 and 1 °C for 30 s in a Nalgene microtube cooler container (Dutscher, France) (thermal shock).

#### ITS sequencing

The rRNA ITS2 region was amplified using the ITS86 forward primer 5′GTGAATCATCGAATCTTTGAA 3′ and ITS4 reverse primer 5′TCCTCCGCTTATTGATATGC 3′ [10]. PCR was done on a classical thermocycler (Thermo Hybaid, Thermo Scientific). Purification of PCR products was then performed using the Exosap IT technique (Amersham, GE Healthcare Europe GmbH, Belgium). Bidirectional sequence data were generated after purification using the BigDye terminator sequencing kit (Applied Biosystems, Life Technologies, Belgium). Sequenced products were finally purified using the kit clean Seq Agencourt (Beckman Coulter Life Science). The sequencing was done on the automate ABI 3500/3500XL (Applied Biosystem, Life Technologies). Sequences were edited using the ABI Sequence Scanner V.1.0 software (Applied Biosystems, Life Technologies). Sequences generated by the software were then compared to the CBS database by using BIOLOMICSNET software (http://www.cbs.knaw.nl/collections/BioloMICSSequences.aspx), which comprises several databases including Genbank. Only results that repeated the same identification at least three times and had a similarity score greater than 95% were considered valid.

#### Serotyping PCR

A classical serotyping PCR, which has been designed for *Cn/Cg* species complex, was performed according to the protocol described by Ito-Kuwa et al. [11] Two primer pairs of the LAC1 gene and one primer pair of the CAP64 gene were used.

#### Antifungal susceptibility testing

Determination of Minimal Inhibitory Concentration (MIC) was done according to the EUCAST E.Def 7.3.1 procedure [12]. Inoculum suspensions of 0.5 McFarland standard were prepared and diluted 1:10 with sterile distilled water (Sensititre tm demineralized water, Thermo Scientific, USA). The final concentration range was 0.008–8 mg/L for amphotericin B (after inoculation) and 0.06–64 mg/L for 5-flucytosine and fluconazole. The reading of the MIC50 value (drug concentration resulting in inhibition of 50% of microorganisms) for 5-flucytosine and fluconazole, and MIC90 for amphotericin B, was done according to the described recommendations using a visual and automated reading at 405 nm with a Multiscan FC spectrophotometer (Thermo Scientific, MA, USA). *Candida parapsilosis* ATCC 22019 and *Candida krusei* ATCC 6258 were used as the quality control strains for the tests. The interpretation criteria for amphotericin B were those defined in EUCAST (European Committee on Antimicrobial Susceptibility Testing) version 10.0 Breakpoint tables: susceptible, MIC ≤ 1 mg/L; Resistant, MIC > 1 mg/L. Being not defined in the EUCAST tables, the interpretation of fluconazole and 5-flucytosine was based on the criteria published by the Clinical Laboratory Standards Institute (CLSI) as follows: for fluconazole, sensitive if MIC ≤ 8 mg/L; dose-dependent sensitive if MIC between 16 and 32 mg/L; resistant if MIC ≥ 64 mg/L; for flucytosine, sensitive if MIC ≤ 4 mg/L; intermediate if MIC between 8 and 16 mg/L; resistant, if MIC ≥ 32 mg/L [13, 14].

### Statistical analysis

The analysis were carried out using R-Cmdr version 2.6-1 (R Foundation for Statistical Computing, Vienna, Austria). Missing data were considered completely random and the available data were analyzed. The continuous variables were summarised as mean ± standard deviation and compared using Student’s t-test. The proportions and their respective 95% confidence intervals were calculated for the categorical data. The main outcome variable was the “species of *Cryptococcus* identified”, which was compared to other variables of the same type using the Pearson Chi-square test or the Fisher exact test if the expected values were less than 5. All tests were two-tailed and a *p* < 0.05 was considered statistically significant.

## Results

### Study population and clinical data

Of the 29 patients included for cryptococcal meningitis with positive culture on CSF, six (20.7%; 95% CI 6.9–34.5%) were infected by non-*neoformans* and non-*gattii Cryptococcus* species. In five cases, it was *C. curvatus*, and in one case *C. laurentii.*

The demographic and clinical characteristics of patients infected by each group species are presented in Table 1. Compared to *Cc/Cl* infected patients, *Cn* infected patients were most susceptible to headache on admission (100% vs 50%, *p* = 0.005). Remarkably, the following neuromeningeal signs were only found in *Cn* infected patients: consciousness disorders (47.8%), meningeal signs (30.4%), cognitive deficits (26.1%), and convulsions (17.4%). The distribution of age, sex, and marital status in the two groups of patients were similar. While all patients infected with *Cn* were in the terminal stage of HIV infection, 20% of patients with *Cc/Cl* were classified in stage III, before cryptococcosis diagnosis has been established (not significant). Approximately 18.3 and 19.3 days were the average time elapsed between the appearance of the first symptoms and the diagnosis of meningitis in the patients’ group with *Cn* versus *Cc/Cl* meningitis, respectively. Comparison of clinical features in *Cn* and *Cc/Cl*-infected patients respectively showed marked differences in fever (78.0% vs 33.3%), weight loss (66.9% vs 33.3%), dizziness (17.4% vs 16.8%), hemiplegia (0% vs 16.7%), neck pain (4.3% vs 0%) and facial paralysis (0% vs 16.7%).

**Table 1:** Demographic and clinical characteristics of HIV-infected patients with *C. neoformans* versus *C. curvatus/C. laurentii* meningitis

Variable	*Cryptococcal* species		*p*
	*C. neoformans* n^a^ (%)^b^	*C. curvatus/C. laurentii* n^a^ (%)^b^	
Demographic characteristics			
Age, mean (year) ± SD	36.7 ± 11·3	38.3 ± 9.1	0.1
Female sex	15 (65.2)	4 (66.7)	1
Marital status			0.56
Single	8 (34.8)	3(60.0)	
Married/free union	10 (43.5)	2 (40.0)	
Divorced/separated/widowed	5 (21.7)	0	
Clinical stage of HIV (WHO)			0.19
Stage III	0	1 (20.0)	
Stage IV	21 (100)	4 (80.0)	
Antiretroviral therapy (ART)	20 (87.0)	3 (50.0)	0.08
Clinical signs			
Headache	23 (100)	3 (50.0)	0.005
Fever	18 (78.0)	2 (33.3)	0.05
Weight loss	16 (69.6)	2 (33.3)	0.16
Disorder of consciousness	11 (47.8)	0	0.05
Meningeal signs	7 (30.4)	0	0.28
Memory impairment	6 (26.1)	0	0.29
Convulsions	4 (17.4)	0	0.55
Dizziness	4 (17.4)	1 (16.7)	1
Visual disturbances	0	2 (33.3)	0.03
Hemiplegia	0	1 (16.7)	0.2
Neck pain	1 (4.3)	0	1
Facial paralysis	0	1 (16.7)	0.2
Mean temperature (°C) ± SD	36.8 ± 0.8	36.1 ± 1.3	0.08
Duration of symptoms	18.3 ± 14.7	19.3 ± 9.4	0.8

^a^With available data

^b^Percentage of columns calculated for each group

### HIV biological data of the patients

The HIV biological analysis results of the patients are presented in Table 2. The mean CD_4_ cell count was 45.6 cells/µL in the *Cn* infected patients, which was significantly lower than in the *Cc/Cl* group (129.8 cells/µL, *p* = 0.02). While 50% of *Cn* meningitis patients had a moderately high viral load (10^3^–10^5^ copies/mL), the only viral load available for the patients infected with *Cc/Cl* was very high (> 10^5^ copies/mL).

**Table 2:** HIV biological data of patients with meningitis due to *C. neoformans* versus *C. curvatus/C. laurentii*

Variable	*Cryptococcal* species		*p*
	*C. neoformans* n^a^ (%)^b^	*C. curvatus/C. laurentii* n^a^ (%)^b^	
CD_4_ (cells/µL) ± SD	45.6 ± 40.8	129.8 ± 142.1	0.02
Viral load (copies/mL)			0.6
Undetectable (< 40)	1 (25.0)	0	
Low (40–10^3^)	1 (25.0)	0	
High (10^3^–10^5^)	2 (50.0)	0	
Very high (≥ 10^5^)	0	1 (100)	

^a^With available data

^b^Percentage of columns calculated for each group

### CSF analysis characteristics

#### CSF analysis characteristics patients

**Fig. 1:**
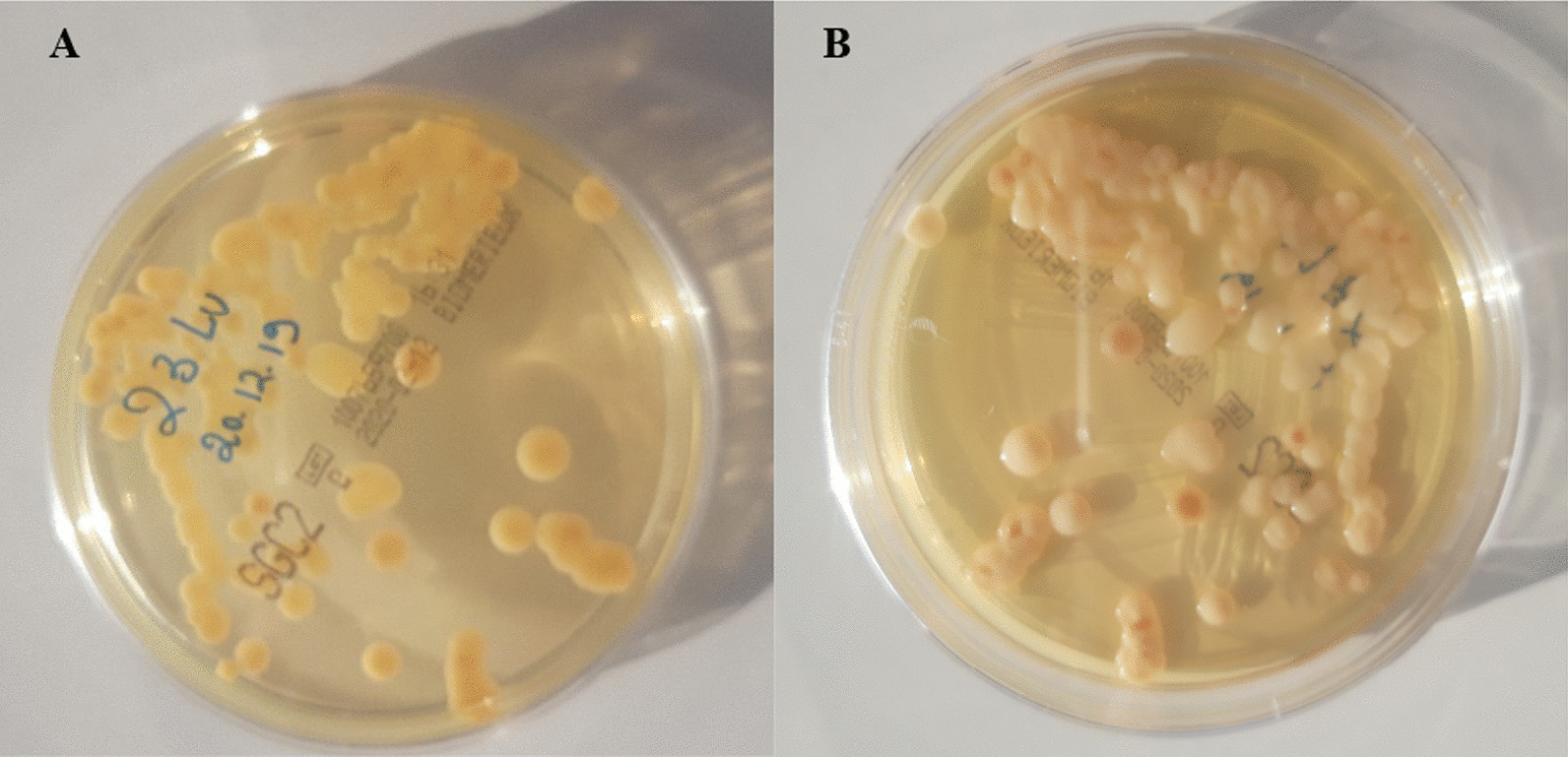
Beige mucoid colonies with reddish hues of *C. curvatus* on SDA-Chloramphenicol after 48-h incubation at 30 °C is shown from the top (**A**) and the bottom (**B**) of the plate

All patients infected with *Cc/Cl* had clear CSF versus 13% of cloudy appearance in *Cn* group patients. In both groups, the majority of patients had a very high opening pressure during a lumbar puncture (75% vs 66.6%, *Cn* and *Cc/Cl* respectively). Approximately 90% of patients with *Cn* meningitis had significantly low glycorrachia (< 50 mg/dL) compared to 20% of *Cc/Cl* infected patients (*p* = 0.01). Accordingly, the mean value of glycorrachia was lower in the *Cn* infected group (46.7 ± 11.2 mg/dL) than in the *Cc/Cl* group patients (66.1 ± 22.5 mg/dL, *p* = 0.04). Sixty per cent of patients infected with *Cc/Cl* had a positive qualitative proteinorachia (Pandy test), while 56.2% of patients infected with *Cn* had a negative test (*p* = 0.6). No significant difference was noted between the mean cytorrachia in the *Cn* patients group versus those infected with *Cc/Cl*. Nearly 33.3% of *Cn* infected patients had a predominantly lymphocytic cytorrachia compared to 50% in the other group. While all the study cases had positive cultures (100% in both groups), three *C. curvatus* strains had atypical presentation with reddish hues within the colonies (Fig. 1). Only 18 *Cn* (out of 23) and two *Cc/Cl* (out of six) samples were positive on direct India ink staining. Similarly for the CrAg test in CSF, 95.7 vs 66.7% were positive in each group, *Cn* and *Cc/Cl* respectively. CSF analysis characteristics are shown in Table 3.

**Table 3:** CSF analysis characteristics of HIV-infected patients with *C. neoformans* versus *C. curvatus/C. laurentii* meningitis

Variable	*Cryptococcal* species		*p*
	*C. neoformans* n^a^ (%)^b^	*C. curvatus/C. laurentii* n^a^ (%)^b^	
The clear appearance of CSF	20 (87.0)	6 (100)	1
Mean opening pressure (cm of water)	30.0 ± 7.6	28.3 ± 7.6	1
CSF opening pressure cm of water)			1
Normal (< 20)	1 (12.5)	0	
High (20–30)	1 (12.5)	1 (33.3)	
Very high (≥ 30)	6 (75.0)	4 (66.6)	
Glycorrachia (mg/dL) ± SD	46.7 ± 11.2	66.1 ± 22.5	0.04
Glycorrachia (mg/dL)			0.01
Low (≤ 50)	9 (90.0)	1 (20.0)	
Normal (50–60)	0	1 (20.0)	
High (≥ 60)	1 (10.0)	3 (60.0)	
Positive pandy	7 (43.8)	3 (60.0)	0.63
Cytorrachia (cells/mm^3^) ± SD	41.52 ± 51.6	15.8 ± 23.5	0.25
Lymphocytic cytorrachia	5 (33.3)	1 (50.0)	1
Positive culture on CSF	23 (100)	6 (100)	1
Positive India ink on CSF	18 (78.3)	2 (33.3)	0.06
CrAg present on CSF	22 (95.7)	4 (66.7)	0.09

^a^With available data

^b^Percentage of columns calculated for each group

#### Species identification

All strains (100%) of *Cn* were identified by ITS sequencing and then by mass spectrometry. In comparison, 20% of the *Cc/Cl* strains were not identified by mass spectrometry although they were all identified by ITS sequencing. Between the same species, slight differences in ITS sequences were noted among both *Cn* and *Cc* isolates. Besides *Cl* isolate which was unidentified by MALDI-TOF MS, one *Cc* isolate was also unidentified, and the same isolate had a slightly different post multiplex PCR electrophoretic profile than others of the same species, namely the absence of the 1100 pb band (lane 5).

**Fig. 2:**
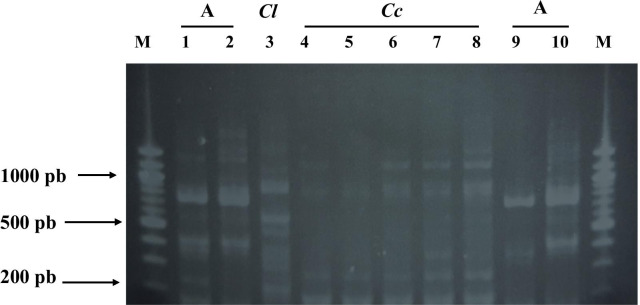
DNA fragments of *Cn* and *Cc/Cl* isolates obtained after multiplex PCR amplification of LAC1 and CAP64 genes. Lanes 1, 2, 9 and, 10, *Cn* serotype A profile. Lane 3, isolate of *Cl* and lanes 4–7 and 8, isolates of *Cc*. M, molecular weight marker. The *Cc/Cl* isolates thus gave DNA bands of different sizes and numbers from the reference profiles. While the *Cn* isolates had given the characteristic major band of serotype A of about 760 bp and a small band of 420 bp, the *Cc* isolates had the first band of about 1100 bp and a second of 800 bp, and the *Cl* isolates had a band of about 900 bp and another of about 600 bp, profiles that don’t match with those of different serotypes of the *Cn/Cg* complex

The difference in MALDI-TOF MS identification between the two groups was not significant; although the spectral profiles were very different. By serotyping multiplex PCR, the *Cc/Cl* strains were not identifiable, whereas the *Cn* strains were all identified as serotype A. The *Cc/Cl* strains had an electrophoretic profile that was not comparable to the reference profile of the *Cn* species commonly serotyped by this technique (Fig. 2). Apart from this, four out of five *Cc* isolates had a similar electrophoretic profile and one was slightly different from the others. The difference in serotype identification was statistically significant in both groups (*p* < 0.0001). The comparison of identification results is presented in Table 4.

**Table 4:** Identification of *Cn* versus *Cc/Cl* by MALDI-TOF MS and PCR serotyping compared to ITS sequencing

Variable	Cryptococcal species^a^		*p*
	*C. neoformans* n (%)	*C. curvatus/C. laurentii* n (%)	
MALDI-TOF MS			0.1
* Cryptococcus* spp.	23 (100)	4 (80)	
Not identified	0	2 (20)	
Multiplex PCR serotyping			< 0.0001
Serotype A	23 (100)	0	
Non identifiable serotype	0	6 (100)	

^a^ITS sequencing identification

### Antifungal susceptibility of patients’ strains

**Fig. 3:**
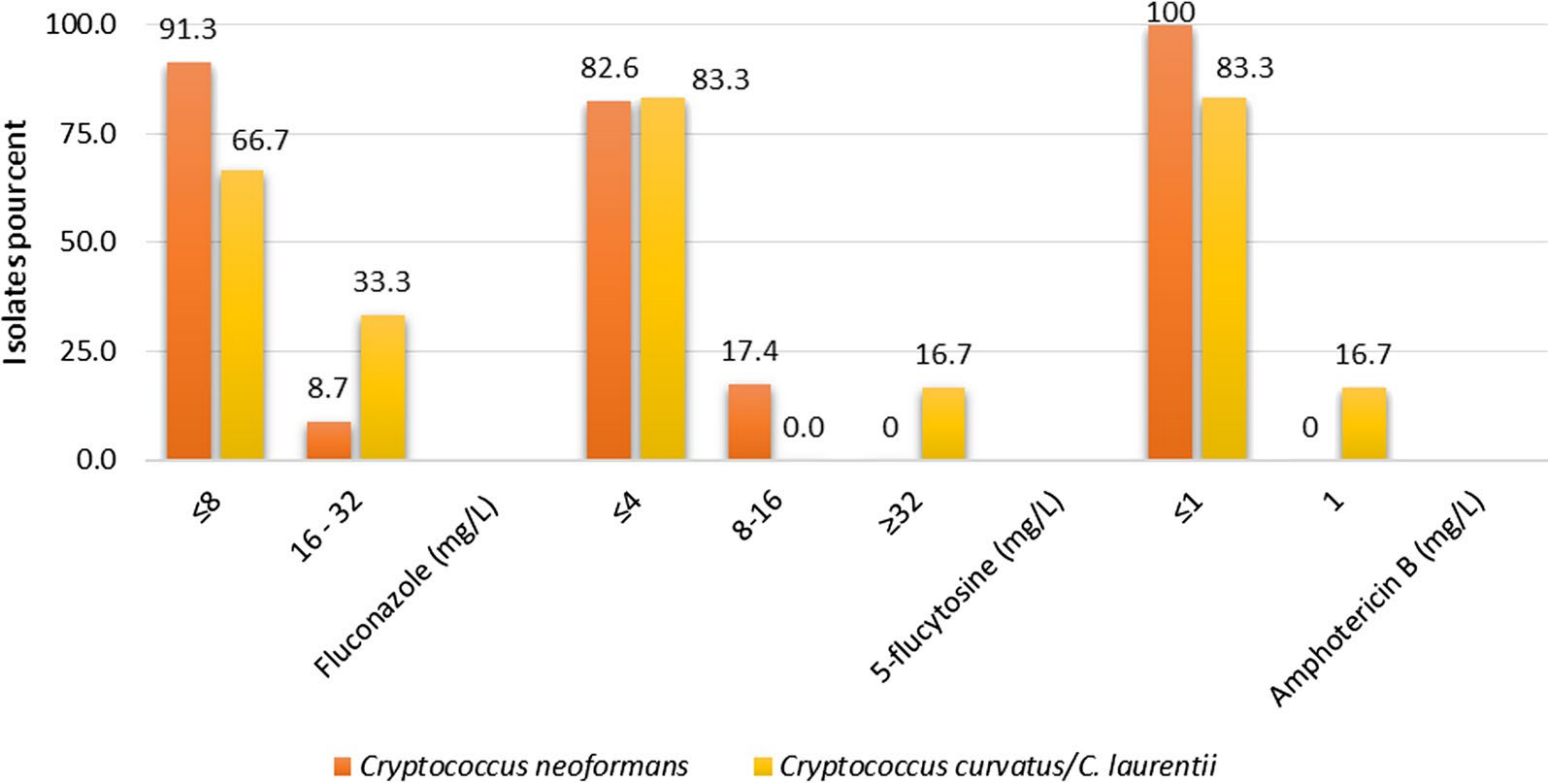
Comparative susceptibility of *Cn* versus *Cc/Cl* strains to usual antifungal agents by EUCAST procedure

About 91.3% of *Cn* group strains (21 of 23) had a MIC ≤ of 8 mg/L for fluconazole, compared to 66.7% in *Cc/Cl* group (four of six). In both groups, the proportion of strains susceptible to 5-flucytosine were almost similar (82.6% and 83.3%, *Cn* and *Cc/Cl* strains respectively) and one strain in the *Cc/Cl* group was resistant (16.7%). While all *Cn* strains were sensitive to amphotericin B, 16.7% of *Cc/Cl* strains were resistant. The only *Cl* isolate that was unidentified by MALDI-TOF MS was resistant to both flucytosine and amphotericin B. In vitro susceptibility profile of strains against each of the three antifungal agents tested was not statistically different in the two groups (Fig. 3).

### Treatment and outcome

Table 5 shows the outcome of *Cryptococcus*-infected patients after treatment by antifungal agents. Treatment with liposomal amphotericin B (3 mg/kg/day for 7 days) combined with 5-flucytosine (100 mg/kg taken four times daily for 7 days) in the induction phase, followed by fluconazole (800 mg/day for 8 weeks) for the consolidation phase was administered to 95.7% of patients who were hospitalized after MC diagnosis. One (33.3%) of the *Cc/Cl* infected patients was treated with fluconazole alone (800 mg/days) due to lack of meningeal location confirmation. Patients infected with *Cc/Cl* remained hospitalized for 12.4 vs 9.9 days in the *Cn* group (not significant). Thirteen out of 23 *Cn* group patients (56.5%) had a pejorative clinical course (death, status quo, and discharge against medical advice or transfer due to complications) versus 16.7% in the *Cc/Cl* group (not significant).

**Table 5:** Treatment and outcome of patients with *C. neoformans versus C. curvatus/C. laurentii* meningitis

Variable	Cryptococcal species		*p*
	*C. neoformans* n^a^ (%)^b^	*C. curvatus/C. laurentii* n^a^ (%)^b^	
Scheme of antifungal agents			0.09
AFF^c^	22 (95.7)	4 (66.7)	
Fluconazole	0	1 (33.3)	
Average hospital stay (day)	9.9 ± 6.01	12.4 ± 5.27	0.65
Therapeutic outcome			0.16
Good^d^	10 (43.5)	5 (83.3)	
Bad^e^	13 (56.5)	1 (16.7)	

^a^With available data

^b^Percentage of columns calculated for each group

^c^Amphotericin B + 5-flucytosine + fluconazole

^d^Recovery and discharge from hospital

^e^Death, status quo, discharge against medical advice or transfer due to complications

## Discussion

Cryptococcal meningitis has been described as an opportunistic infection in HIVIP, mainly caused by the *Cn/Cg* species complex [15, 16]. However, non-*neoformans* and non-*gattii Cryptococcus* species have recently been isolated in cryptococcosis both in immunosuppressed and immunocompetent patients. *C. curvatus* and *C. laurentii* are among these species [3, 6, 17]. While *C. laurentii* has already been associated with bloodstream, neuromeningeal, pulmonary, cutaneous, and peritoneal infections [3, 4, 18–20], *C. curvatus* has very rarely been implicated in human infections, mainly in peritoneal and myeloradicular infections [5–7].

Of the 29 *Cryptococcus* spp. meningitis patients with positive culture included in the present study, six (20.7%, 95% CI 6.9–34.5%) had non-*neoformans* and non-*gattii Cryptococcus* species, including five cases of *C. curvatus* and one case of *C. laurentii.* To our best knowledge, this study is the first to compare the biological and clinical characteristics of *Cryptococcus* spp. meningitis induced by these two species groups in the sub-Saharan African region. Globally, 79.3% were identified as *Cn* and 20.7% as *Cc/Cl*. A similar prevalence of non-*neoformans* and non-*gattii Cryptococcus* infections was reported in three states of the United States of America (USA) in 2020 and during the same study, *C. laurentii*, *C. liquefaciens,* and *C. magnus* were considered as pathogenic [21].

Patients with *Cn* were most susceptible to headache on admission than patients with *Cc/Cl* and most neuromeningeal signs were also found preferentially in *Cn* patients. The distribution of these signs could suggest a more virulent trait of *Cn* strains compared to *Cc/Cl* strains. The main virulence factors of the *Cryptococcus* genus i.e. presence of the polysaccharide capsule, resistance to high temperatures (> 37 °C), and the activity of the laccase enzyme, have been identified for the *Cn* complex as well as for the non-*neoformans* and non-*gattii Cryptococcus* species. However, the laccase enzyme activity was described as lower for non-*neoformans* and non-*gattii Cryptococcus* species compared to that of the *Cn* [7]. This could explain the more severe clinical presentation of the *Cn* group patients compared to *Cc/Cl* group, as described also by Cano et al. [21].

As mentioned above, most species of the genus *Cryptococcus* possess a polysaccharide capsule and share the same antigenic determinants with minor differences, and are therefore likely to be detected by India ink staining and/or have antigenic activity detectable by diagnostic tests [22]. Nevertheless, identification of *Cryptococcus* species still requires strain culture and PCR assays. As described in the literature [7], the positivity rate of India ink detection in the present study was higher in the *Cn* group than in the *Cc/Cl* group, similarly for CSF detection of cryptococcal antigens which had a much higher positivity rate in the *Cn* group. The partial expression of some virulence enzymatic factors in non-*neoformans* and non-*gattii Cryptococcus* species as described by K. Ferreira-Paim et al., and mentioned earlier in this manuscript could explain the low reactivity of *Cc/Cl* capsular antigens to the CrAg assay, which was developed for the detection of *Cn/Cg* species complex antigens. Given the increase in meningitis cases due to non-*neoformans* and non-*gattii* species worldwide, it is useful that the reactivity of these species to commercially available CrAg assays be established in subsequent larger studies [23].

For culture-positive samples with negative CrAg results, the antigen excess zone (prozone phenomenon) may be the explanation. Dilution of the tested sample could have resolved this discordance.

While *Cn* strains were easily identified by MALDI-TOF MS, ITS sequencing, and multiplex PCR serotyping used during the study, only four *Cc/Cl* strains (out of six) were identified by MALDI-TOF MS and all of them had a different profile from the reference agarose gel profile after multiplex PCR. The results of the MALDI-TOF MS identification were only 80% conclusive for the *Cc/Cl* strains. In the remaining cases, it was either an identification with a bad score or an outright wrong identification. As the spectrum generated during sample analysis is compared with the spectra in the manufacturer’s database to establish a match, a limited panel of spectra or the absence of spectra of a microbial species in the database could result in a failed species identification. As both species are present in the database used, BD 8326 Bruker, an extraction process using ethanol, formic acid, and acetonitrile might have been necessary to improve the results because of the “big shell” of *Cryptococcus* spp. The non*-neoformans*/non*-gattii* shell could be more refractory than that of *Cn*. For its part, the determination of serotypes of *Cc/Cl* strains by classical multiplex PCR targeting LAC1 and CAP64 genes was not possible. Based on its initial application, this PCR was designed to characterize strains of the *Cn/Cg* species complex. Given the superiority of the laccase enzyme activity of the *Cn/Cg* complex over that of non-*neoformans* and non-*gattii* species as described above, these results could be partially explained [24].

Although some peritonitis due to non-*neoformans* and non-*gattii Cryptococcus* species has been cured by early removal of the catheter without antifungal treatment, others require more intensive treatment because of the fluconazole and 5-flucytosine resistance associated with these isolates [3, 25].

In the present study, 33.3% of the *Cc/Cl* strains had moderately high MICs to fluconazole (16–32 mg/L) compared to 8.7% in the *Cn* group. The proportion of *Cc/Cl* strains resistant to 5-flucytosine and fluconazole is evaluated between 50 and 100% in other studies and higher than in *Cn/Cg* complex, which is more marked than what we could observe [4, 7, 20]. One strain (16.7%) of the *Cc/Cl* group, *C. laurentii*, was resistant to amphotericin B which is known as the most effective antifungal agent in the management of *Cryptococcus* infections by all species [3, 26]. This same strain was also resistant to 5-flucytosine (16.7%).

All patients with *Cn* meningitis received antifungal agents according to WHO recommendations and guidelines, MSF protocol [27]. In contrast, one patient in the *Cc/Cl* group was treated with fluconazole alone although the strain was susceptible in vitro. Despite this, the patients’ therapeutic outcome was not significantly different in the two groups. Patients were selected in MSF-supported clinics where the whole management process is codified. Thus, patients for whom the *Cryptococcus* identification was provided by the study a few weeks after sample collection were not treated based on this identification. Consequently, 4.3% of patients in the *Cn* group versus 33.3% of patients in the *Cc/Cl* group were not treated with antifungals. The mean length of hospital stay for patients was 12.4 versus 9.9 days with the usual tri-antifungals, *Cc/Cl,* and *Cn* groups respectively. Longer hospital stays (60 days) have been described for *C. albicans* meningitis on amphotericin B [28].

## Conclusions

*Cn* meningitis is clinically more severe than that caused by the *Cc/Cl* complex due to the high virulence of this species. Accordingly, some biological parameters are more altered in *Cn* infection than *Cc/Cl*, on one side. The routine biological detection and molecular identification of *Cc/Cl* strains are more delicate than *Cn*. Plus, *Cc/Cl* strains require high antifungal MICs than *Cn* in vitro, on the other side. That is why *Cc/Cl* complex should be considered in HIVIP management with meningitis.

## Data Availability

All data analysed and generated in this study are available from the corresponding author on reasonable request.

## Abbreviations

*Cc/Cl*: *Cryptococcus curvatus/C. laurentii*
CHME de NGABA: Centre Hospitalier Mère et Enfant de NGABA
CHRB 1^er^: Centre Hospitalier Roi Baudouin 1^er^
CME LUYINDU: Centre Médical et Evangélique LUYINDU
*Cg*: *Cryptococcus gattii*
*Cn*: *Cryptococcus neoformans*
CLSI: Clinical Laboratory Standards Institute
CrAg: Cryptococcal Antigen
CSF: Cerebral Spinal Fluid
DNA: DesoxyriboNucleic Acid
EUCAST: European Committee on Antimicrobial Susceptibility Testing
HCCA: Cyano-4-hydroxycinnamic
HIVIP: HIV-Infected Patients
ITS: Internal Transcribed Spacer
LFA: Lateral Flow Antigen
MALDI-TOF MS: Matrix-Assisted Laser Desorption Ionization Time-Of-Flight Mass Spectrometry
MC: Meningeal Cryptococcosis
MIC: Minimum Inhibitory Concentration
MSF: Médecins Sans Frontière
rRNA: Ribosomic RiboNucleic Acid
SDA-C: Sabouraud Dextrose Agar-Chloramphenicol
